# Maturation of human early-minted blood antibody-secreting cells is coupled with increased IgG secretion rates

**DOI:** 10.3389/fimmu.2025.1644102

**Published:** 2025-09-02

**Authors:** Doan C. Nguyen, Ian T. Hentenaar, Monica Cabrera-Mora, Shuya Kyu, Andrea Morrison-Porter, Natalie S. Haddad, Joel Andrews, Danielle Roberts, Sagar Lonial, Ignacio Sanz, F. Eun-Hyung Lee

**Affiliations:** ^1^ Division of Pulmonary, Allergy, Critical Care, and Sleep Medicine, Department of Medicine, Emory University, Atlanta, GA, United States; ^2^ Department of Hematology and Medical Oncology, Winship Cancer Institute, Emory University, Atlanta, GA, United States; ^3^ Division of Rheumatology, Department of Medicine, Emory University, Atlanta, GA, United States; ^4^ Lowance Center for Human Immunology, Emory University, Atlanta, GA, United States

**Keywords:** immunoglobulin, antibody-secreting cell, plasma cell, blood, bone marrow, secretion, survival, maturation

## Abstract

Plasma cells are known antibody-secreting factories with immunoglobulin (Ig) transcripts that increase as the cell matures into a long-lived plasma cell (LLPC) in the bone marrow (BM). Whether the Ig secretion rates among human antibody-secreting cells (ASC) are homogeneous or BM LLPC are capable of secreting more antibodies per cell compared to early-minted blood ASC remain unclear. Here, we use bulk and single cell cultures in a novel *in vitro* BM mimetic survival system to measure the IgG secretion rates of human ASC. We find that the mature BM ASC produce more IgG molecules per cell compared to immature, early-minted blood ASC. Furthermore, these blood ASC can mature into LLPC phenotypes in culture, and we show that ASC on day 7 secrete more IgG per cell than the input ASC from day 0. Thus, as human ASC mature, they increase the number of Ig transcripts and result in higher Ig secretion. These results also demonstrate that the mature ASC in the BM have higher Ig secretion rates compared to early-minted blood ASC.

## Introduction

Human antibody-secreting cells (ASC) are now well recognized to be heterogeneous, consisting of early-minted ASC in the blood and intermediate and mature plasma cells in the bone marrow (BM) (1–3). In the blood, immature ASC can also be quite heterogeneous (4) while in the BM, ASC can be transcriptionally classified as short-lived, intermediate, and long-lived plasma cells (LLPC) that have the potential to survive indefinitely (5–7). Phenotypically, human BM ASC are heterogeneous with PopA exemplifying an immature subset, PopD (LLPC) representing the most mature one, and PopB as a diverse group that consists of intermediate phenotypes (5, 8, 9). Although difficult to study *in vivo*, the model of human ASC maturation into a LLPC was initially described with the use of a novel *in vitro* BM mimetic system (10, 11). This study (11) illustrates that maturation takes place upon arrival to the BM niche and that early-minted ASC from the blood can undergo further maturation in the BM niche conditions, which is mimicked, at least in part, by our culture system (4, 5, 8, 10). For clarity, the term ASC is used to refer to all Ig-secreting cells, which include early-minted blood ASC (plasmablasts) (3) and mature ASC known as plasma cells that can contain the LLPC subset.

As a protein secreting factory, a human ASC can produce massive amounts of immunoglobulins (Ig): 100-10,000 molecules per cell per second or 2–220 pg/cell/day (pg/c/d) (12–16). Studies reported a wide range of Ig secretion rates with some as high as 3,334 pg/c/d (17), while ASC differentiated *in vitro* from human B cells secrete less at 20–140 pg/c/d (18, 19), and malignant myeloma cells have even lower rates (20). These Ig secretion rates were thought to be influenced by both extrinsic and intrinsic factors (11, 21) since mature ASC have expansion of subcellular secretory network (the Golgi and ER) and organelles important in metabolism (mitochondrial mass) which prepare the cells to specialize in Ig secretion (11, 21). In addition, we found increasing Ig transcripts from blood to BM ASC, with the most mature LLPC subset containing the highest number of Ig transcripts (8). Whether these differences ultimately translate into higher Ig secretion rates remain unexplored.

Since ASC rapidly die *ex vivo*, we use a cell-free specialized culture system derived from factors within the human BM microniche that enables survival of ASC to measure Ig secretion rates (10). Since this system also mimics the BM microniche, we can follow the maturation process of blood ASC into a mature BM ASC phenotype (11). Here, we show that mature BM ASC secrete more Ig per cell compared to early-minted blood ASC, illustrating the heterogeneity of Ig secretion rates. As blood ASC mature in this BM mimetic system, they begin to secrete more Ig over time, demonstrating the importance of the maturation process for increased Ig secretion.

## Materials and methods

### Human subjects

We enrolled 69 healthy adults (aged 23–65 years) for peripheral blood samples collected 6–7 days after vaccination with influenza, Tdap (tetanus, diphtheria, and acellular pertussis), hepatitis A, hepatitis B, shingles, HPV, or COVID-19. We also obtained 32 BM aspirates from healthy adults (aged 21–68 years, with 13 men (41%) and 19 women (59%)). Samples were collected during July 2014-March 2024. All samples were fresh and no frozen samples were used. All research was approved by the Emory University Institutional Review Board and written informed consent was obtained from all subjects.

### ASC purification and a human myeloma cell line

Mononuclear cells from peripheral blood and BM aspirate samples were isolated, enriched, and stained as previously described (5, 10). Cells were sorted on a BD FACSAria II as (3, 5, 10): blood ASC (IgD^-^CD27^hi^CD38^hi^) (**Supplementary Figure 1a**), BM PopA (CD19^+^IgD^-^CD38^hi^CD138^-^), BM PopB (CD19^+^IgD^-^CD38^hi^CD138^+^), and BM PopD (CD19^-^IgD^-^CD38^hi^CD138^+^) (**Supplementary Figure 1b**). The human myeloma cell line, ARH-77, was purchased (ATCC).

### *In vitro* BM mimetic cultures

ASC were cultured for one day or up to 7–8 days in the BM mimetic system, which consists of the BM mesenchymal stromal cell (MSC) survival medium and in hypoxia (e.g., 2.5% O2 and 5% CO2) at 37°C supplemented with 200 ng/mL APRIL (R&D Systems) (10). ARH-77 cells were handled as per recommendations (3).

### ELISpots, ELISA, and multiplex bead binding assays

We quantified total and vaccine-specific IgG ASC using ELISpots, as previously described (3, 9, 10) (**Supplementary Figure 2**). We measured secreted IgG by MBBA (9) or by ELISA. For the ELISA, 96-well flat-bottom ELISA microplates (Nunc/Corning) were pre-coated overnight at 4°C with goat anti-human IgG or specific vaccine. Plates were then washed using a microplate washer (Biotek) and nonspecific binding was blocked with SuperBlock Blocking Buffer (Thermo Fisher), followed by washing. Culture supernatants and the standard protein molecules or the monoclonal antibody (mAb) standards were then loaded and plates were incubated, followed by washing. Alkaline phosphatase–conjugated secondary antibody was then added and plates were incubated, followed by washing. The amount of bound secondary antibody was visualized with an enzymatic color reaction using BluePhos Microwell Phosphatase Substrate System (KPL). Light absorbance was measured at 650 nm using a microplate reader (BioTek). The sensitivity of the assay was ~0.17-0.34 ng/mL. The intra-assay variability of the assay was <12%, as determined by measuring multiple sets of supernatants in two separate runs.

For vaccine-specific IgG capturing, tetanus toxoid Clostridium tetani (Calbiochem) or quadrivalent influenza vaccine 2015-2016 (Fluarix Quadrivalent Influenza Vaccine 2015–2016 Formula; GSK Biologicals) was used. For relative quantitation of the concentrations of total IgG or antigen-specific IgG, standard curves were generated using purified human IgG (ChromePure human IgG, JacksonImmuno Research Laboratories) or mAb standards of anti-tetanus toxin mAb (clone #TetE3; The Native Antigen Company).

### Calculation of the IgG secretion rates

As the amount of total IgG secreted by a single ASC in one day was below the detection limit by our assays, we maintained single-sorted ASC *in vitro* for 7–8 days. For bulk cultures, average IgG concentrations were calculated based on the number of spots and the concentrations of secreted IgG (assuming equal secretion rates both per cell and per day). For single cell cultures, although blood ASC could increase Ig secretion rates with maturation in the cultures, we assumed equal secretion per day and measured the cumulative total IgG amount in the cultures and divided by number of days in culture. Except for day 0, the secretion rates for bulk cultures were calculated as follows: Secretion rates (pg/cell/day) = Amount (pg) of IgG secreted in culture supernatants/(Quantity of IgG spots x Number of days in culture). For single cell cultures, the secretion rates were calculated as follows: Secretion rates (pg/cell/day) = Amount (pg) of IgG secreted in culture supernatants/Number of days in culture.

### On-chip single-cell culture, in-channel IgG capture, and fluorescence semi-quantitation

To overcome apoptosis and ongoing maturation in the 7-day single ASC cultures, we directly assess single ASC secretion rates using a novel microfluidic system to visualize IgG secretion *ex vivo* with high precision (3). Single ASC visualization was performed using the Lightning system (Bruker Cellular Analysis) (3). The instrument captures individual ASC in the act of Ig secretion (3) which develops as fluorescent halos (“blooms”) generated by accumulation of fluorescence from secondary antibodies on the IgG-coated bead upon secreted IgG binding in the channels adjacent to the pens. We loaded blood ASC and BM ASC on the Lightning platform and directly visualized IgG production from a single primary ASC *ex vivo* within 30 minutes (3, 10). Semi-quantitation of relative fluorescent signal intensity of blooms was performed using ImageJ (NIH) (3, 22). Specifically, we calculated the relative corrected total in-channel fluorescence intensity (CTiCF) across an in-channel region of interest by subtracting out the mean fluorescence of background (Reference) readings as follows: CTiCF = Integrated density – (Area of selected region x Mean fluorescence of background readings) (22).

### Statistics

Statistical differences and correlation were evaluated using Excel (Microsoft) and GraphPad Prism (GraphPad Software). *p* values (calculated with Student’s *t-test* (two-tailed unpaired *t-test*)) of ≤0.05 were considered significance.

## Results

### Mature ASC have higher IgG secretion rates

**Figure 1:**
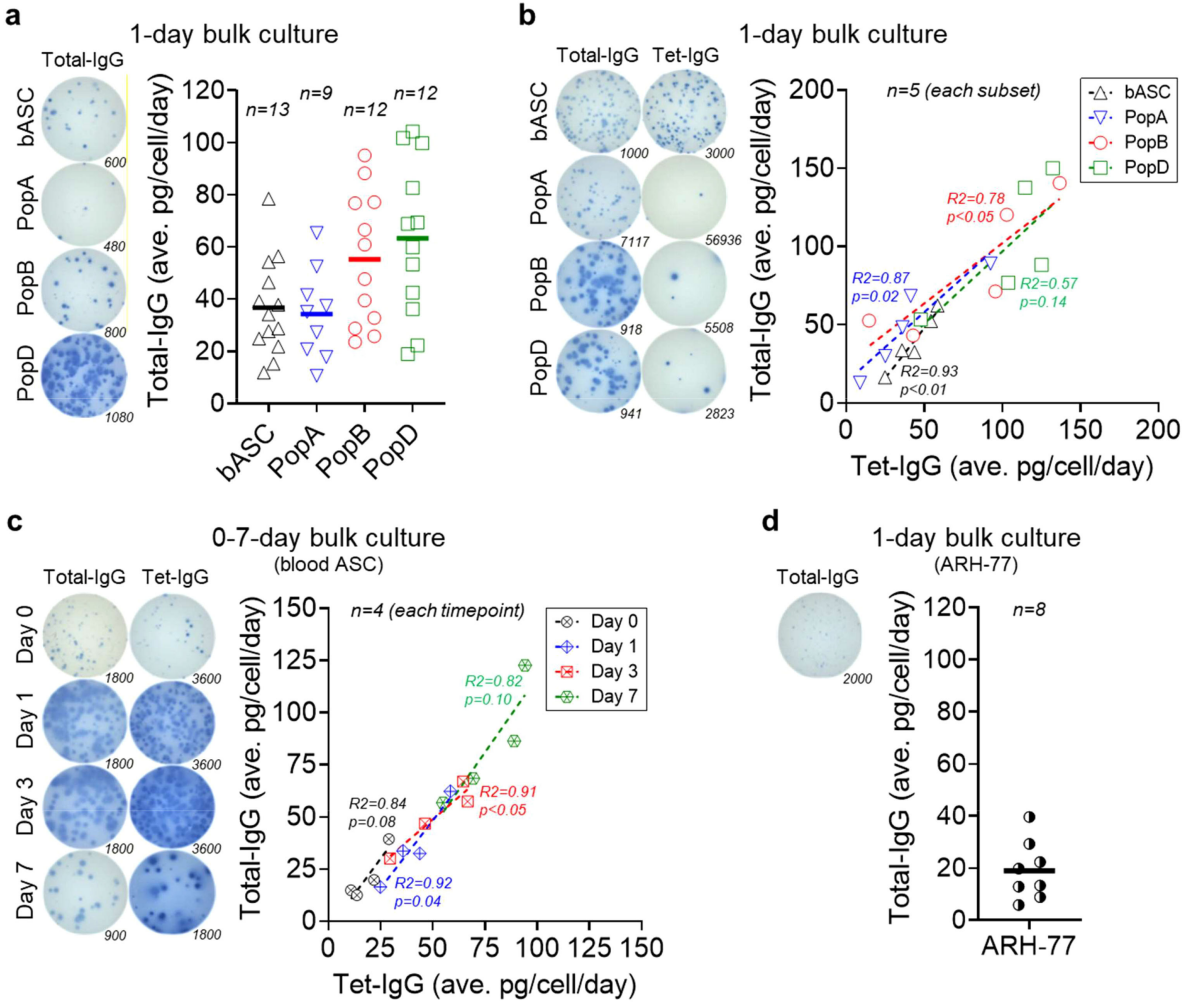
Mature ASC have higher IgG secretion rates by bulk culture interrogation. **(a)** Average total IgG secretion rates by blood ASC and BM ASC maintained in the BM mimetic bulk cultures for 1 day. (*Left*) Representative ELISpot scanned images; numbers indicate the quantity of the input ASC (seeded at day 0). (*Right*) Each symbol represents one experiment. Numbers (n) indicate the quantity of independent biological experiments. **(b)** Average total IgG and Tet-IgG secretion rates by blood ASC and BM ASC maintained in the BM mimetic bulk cultures for 1 day. (*Left*) Representative ELISpot scanned images; numbers indicate the quantity of the input ASC (seeded at day 0). (*Right*) Each symbol represents one experiment (per each subset). Numbers (n) indicate the quantity of independent biological experiments. **(c)** Average total IgG and Tet-IgG secretion rates by blood ASC maintained in the BM mimetic bulk cultures for up to 7 days. (*Left*) Representative ELISpot scanned images; numbers indicate the quantity of the input ASC (seeded at day 0). (*Right*) Each symbol represents one experiment (per each timepoint). Numbers (n) indicate the quantity of independent biological experiments. **(d)** Average total IgG secretion rates by ARH-77 cells maintained in the *in vitro* BM mimetic bulk cultures for one day. (*Left*) A representative ELISpot scanned image; the number indicates the quantity of the input ARH-77 cells (seeded at day 0). (*Right*) Each symbol represents one experiment. The number (n) indicates the quantity of independent biological experiments. For statistical differences among groups (ASC subsets or timepoints) in **(a-c)**, see **Supplementary Tables 1**, **2**, and **4**, respectively. In **(b, c)**, R and *p* values calculated from simple linear regression analysis in GraphPad Prism (GraphPad Software). In **(c)**, blood ASC day 1 data reproduced from four out of five blood ASC experiments shown in **(b)**. bASC, early-minted blood ASC; Tet, tetanus.

We first interrogated production of total IgG in healthy human ASC *ex vivo* from the blood and the BM. In one-day bulk cultures of blood ASC, the average IgG secretion rate is 37 ± 19 pg/c/d, which is similar to PopA (34 ± 17 pg/c/d), but significantly lower than PopB (55 ± 26 pg/c/d) or PopD (63 ± 30 pg/c/d) (**Figure 1a**). While secretion rates by blood ASC or PopA are significantly lower than PopB or PopD, no significant differences exist between PopB vs PopD (**Supplementary Table 1**). Thus, IgG secretion rates increase from blood ASC and early BM ASC (PopA) to mature BM ASC (PopB and PopD). This finding is consistent with high Ig transcript abundance in PopD, the most mature BM ASC subset (8).

### Mature ASC have higher vaccine-specific IgG secretion rates

Single cell transcriptomics of the human BM ASC show tremendous heterogeneity (8). Thus, we next examined the blood ASC from adults on day 6–7 after Tdap vaccination, which is the peak of the vaccine responses, compared to the BM ASC from adults 5–10 years after the Tdap vaccine. We measured if average Tdap vaccine-specific (Tet-) IgG secretion rates differ from total IgG secretion rates in blood and BM ASC in one-day bulk cultures (9). As expected, the frequencies of Tet-IgG ASC are 35 ± 15% of the total IgG ASC in the blood days after vaccination, compared to the Tet-IgG ASC frequences of 0.3 ± 0.2%, 1.2 ± 0.6%, and 2.4 ± 1.1% of total IgG PopA, PopB, and PopD, respectively, in the BM years after the vaccination. We also observe blood ASC and PopA are the lowest secretors: average total IgG of 39 ± 18 pg/c/d or 50 ± 30 pg/c/d, and average Tet-IgG of 43 ± 14 pg/c/d or 41 ± 31 pg/c/d for blood ASC or PopA, respectively (**Figure 1b**). For mature BM ASC, PopB and PopD secrete substantially higher total IgG (average of 86 ± 43 pg/c/d and 101 ± 41 pg/c/d, respectively) and Tet-IgG (average of 78 ± 49 pg/c/d and 105 ± 34 pg/c/d, respectively). In all, there are differences between average total IgG secretion rates of blood ASC vs PopB and blood ASC vs PopD, and between average antigen-specific (Tet-) IgG secretion rates of blood ASC vs PopD and PopA vs PopD (**Supplementary Table 2**). The similar total IgG and Tet-specific IgG secretion rates among the ASC subsets suggest that differences in secretion rates are likely influenced by the maturity of the ASC.

### Blood ASC after *in vitro* maturation have higher IgG secretion rates

Unlike the mouse models, human ASC *in vivo* timestamping is difficult in order to follow early-minted blood ASC into the BM microniches. Therefore, we developed an *in vitro* maturation culture system to observe step-wise programs of early-minted blood ASC in long-lived phenotypes (8, 23). Using this system, we next evaluated if the same immature blood ASC could increase IgG secretion rates after *in vitro* maturation. We measured blood ASC total IgG and Tet-IgG secretion rates at day 0, 1, 3, and 7 in culture. Total IgG and Tet-IgG gradually accumulated in the supernatants (**Supplementary Figure 3** and **Supplementary Table 3**), suggesting cultured blood ASC maintain functionality overtime. Assuming individual blood ASC produce the same Ig amount each day and have equal survival rates within the assessing period, we observe a progressive increase in average total IgG secretion rates: from 22 ± 12 pg/c/d at day 0 to 36 ± 19 pg/c/d at day 1, then 50 ± 16 pg/c/d at day 3, and 84 ± 29 pg/c/d at day 7 (**Figure 1c**). While no significant difference exists between average total IgG secretion rates for day 0 vs day 1, day 1 vs day 3, and day 3 vs day 7, there is significance between day 0 vs day 3, day 0 vs day 7, and day 1 vs day 7 (**Supplementary Table 4**). We also see a similar pattern of progressive increase in average Tet-IgG secretion rates: 19 ± 8 pg/c/d, 41 ± 14 pg/c/d, 52 ± 17 pg/c/d, and 77 ± 18 pg/c/d at day 0, day 1, day 3, and day 7, respectively. Thus, the same blood ASC has higher average total IgG and Tet-IgG secretion rates following *in vitro* maturation. These kinetics experiments of human blood ASC maturation are the first to clearly illustrate these differences.

### ARH-77 myeloma cells have lower IgG secretion rates relative to healthy blood ASC

To understand secretion rates of healthy vs malignant plasma cells, we evaluated a particular myeloma cell line, ARH-77. As shown previously, the size of the IgG ELISpots of this myeloma cell line was much smaller compared to that of healthy blood ASC or BM ASC, suggesting lower secretion rates (3). Corroborating this finding, we show ARH-77 secrete on average 19 ± 11 pg/c/d (**Figure 1d**) which is less than the rates from blood or BM ASC. Appreciating that primary myeloma cells and myeloma cell lines are also very heterogeneous, it is likely myeloma cell secretion rates may be quite variable. Nonetheless, for this particular established myeloma cell line, secretion rates are lower than those of healthy human ASC.

### Mature ASC have higher IgG secretion rates on a single cell basis

**Figure 2:**
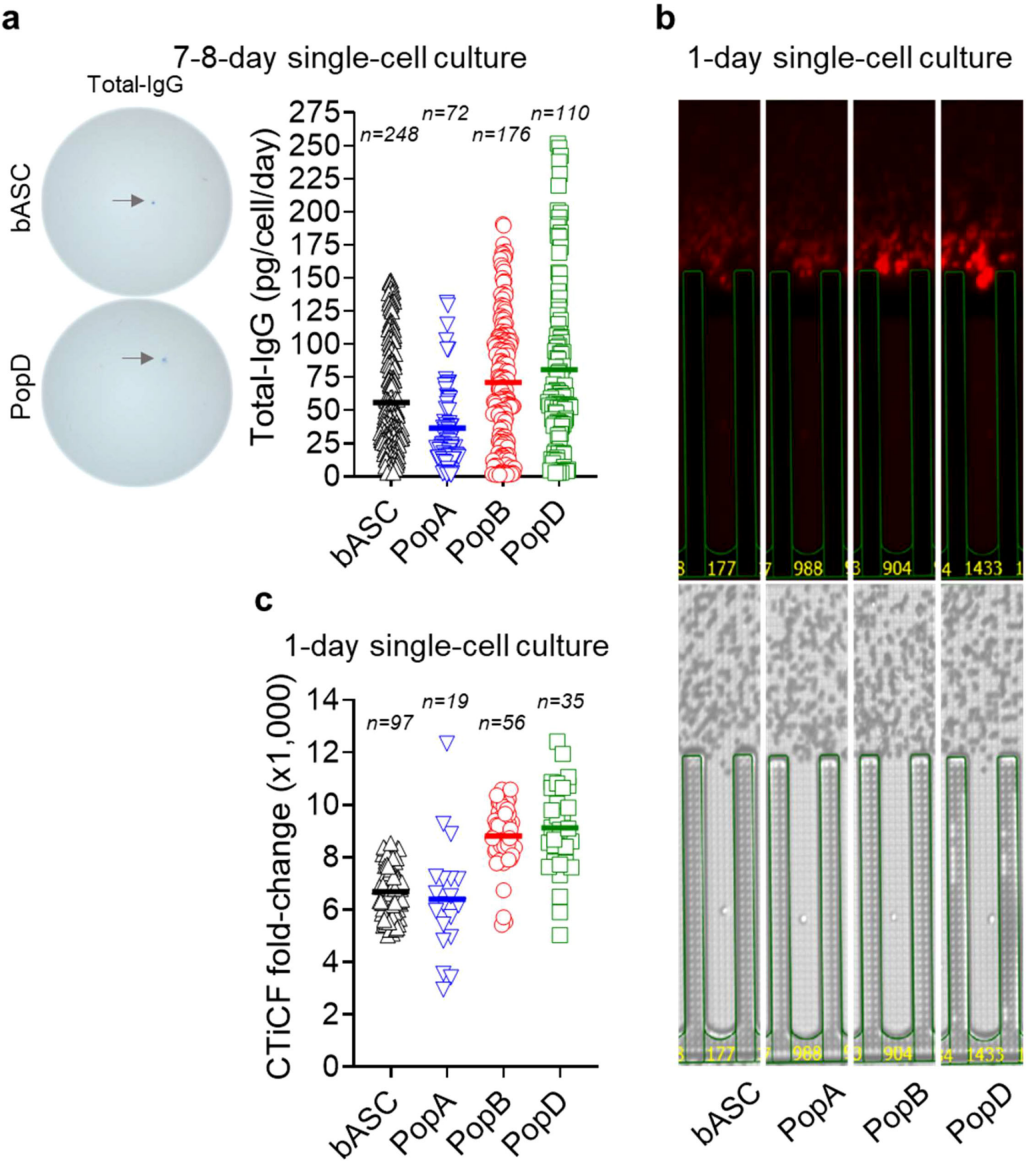
Mature ASC have higher IgG secretion rates by single cell culture interrogation. **(a)** Total IgG secretion rates by blood ASC and BM ASC maintained in the BM mimetic single-cell cultures for 7–8 days. (*Left*) Representative ELISpot scanned images. (*Right*) Each symbol represents one cell. Numbers (n) indicate the quantity of individual cells. **(b)** Representative image series displaying ongoing IgG secretion by a single blood ASC and BM ASC maintained in the BM mimetic single-cell cultures on-chip for one day. (*Lower*) Bright-field image series for direct visualization of a single cell in a (nano)pen. (*Upper*) The act of ongoing IgG secretion into the channel developed as fluorescent halos (“bloom”; captured at the end of an 80-minute cycle). **(c)** CTiCF fold-changes as an indirect comparison of total IgG secretion rates among the four ASC subsets. Each symbol represents one in-pen cell. Numbers (n) indicate the quantity of individual in-pen cells. For statistical differences among ASC subsets in **(a, c)**, see **Supplementary Tables 5** and **6**, respectively. All the four ASC subsets were run on the same chips (for CTiCF comparison) and by all the same capture assays. bASC, early-minted blood ASC.

We next studied if total IgG secretion rates generated from bulk cultures are corroborated by single cell cultures after 7–8 days. We see that on a single cell basis, in blood ASC, total IgG secretion rate is 56 ± 39 pg/c/d, while for BM ASC, total IgG secretion rates are the lowest in PopA (37 ± 30 pg/c/d) and higher in PopB (71 ± 48 pg/c/d) and PopD (81 ± 64 pg/c/d), with no significant difference between PopB vs PopD (**Figure 2a** and **Supplementary Table 5**). Thus, similar to bulk cultures, mature ASC have higher total IgG secretion rates on a single cell basis. Although not statistically significant, single blood ASC surviving in 7-day single cell cultures have a greater variability of IgG secretion rates compared to 1-day bulk cultures, most likely due to ongoing maturation in the cultures even at the single cell level.

We then investigated total IgG and influenza-specific (Flu-) IgG secretion rates in single-sorted blood ASC maintained *in vitro*. Of 192 single-cells, total IgG and Flu-IgG are detected in 165 (~86%) and 114 (~59%), respectively, indicating high plating efficiency. We show a wide range of total IgG and Flu-IgG secretion rates (3–148 pg/c/d and 12–142 pg/c/d, respectively), with average total IgG secretion rates of 48 ± 42 pg/c/d and Flu-IgG secretion rates of 35 ± 40 pg/c/d (**Supplementary Figure 4**). Similar to total IgG and Tet-IgG (**Figure 1b**), we also note similar secretion rates for total IgG and Flu-IgG. Thus, significant differences in secretion rates may be based on the maturity of the ASC and not necessarily the antigen of interest.

### Mature ASC have higher IgG secretion rates by direct single cell visualization

To overcome apoptosis and ongoing maturation in 7-day single cultures, we directly interrogated single ASC secretion rates. Total IgG blooms are captured (**Figure 2b**) and semi-quantitatively calculated as CTiCF fold-changes from the background (3). We show PopB and PopD secrete more IgG per cell compared to blood ASC or PopA, with average CTiCF fold-change by blood ASC, PopA, PopB, or PopD is 6,687, 6,397, 8,809, or 9,120, respectively (**Figure 2c**). Significant fold-change differences (*p*<0.01) exist between blood ASC vs PopB, blood ASC vs PopD, PopA vs PopB, and PopA vs PopD, but not (*p*>0.29) between blood ASC vs PopA or PopB vs PopD (**Supplementary Table 6**). Thus, blood ASC or PopA consistently produces less IgG per cell than PopB or PopD, suggesting increased Ig transcripts (8) lead to increased Ig secretion.

## Discussion

In this study, we show that total IgG and vaccine-specific IgG secretion rates are higher in mature BM plasma cells (PopB and PopD) compared to immature blood ASC and BM PopA, the latter may include new arrivals and cells near death. As a fraction of blood ASC migrate to BM (23–25), the majority of these new arrivals die while some undergo progressive maturation (5, 11, 23, 25). Early studies showed transcriptional differences between BM plasma cells compared to earlier non-BM plasma cells (26, 27). Furthermore, single-cell RNAseq revealed heterogeneity of the human BM plasma cell pool and offered a trajectory analysis with a step-wise maturation process from new arrivals to a *bona fide* LLPC (8). The findings of this study provide significant insights into the functional heterogeneity of human ASC and their maturation-dependent Ig secretion capacities.

A wide range in the IgG secretion rates of human ASC have been previously documented. However, many of the previous studies used *in vitro* differentiated ASC from stimulated memory B cells (18, 28, 29), which may not reflect human *ex vivo* ASC from the blood and BM (3). Other studies were limited to blood ASC *ex vivo (* 14, 17, 30) but did not show a comparison of BM ASC with the same methods. Furthermore, none of the studies compare differences of early-minted blood ASC found with the mature BM ASC that contain the LLPC compartments. Finally, no previous studies show a direct comparison of the IgG secretion rates from human blood and BM ASC at a single cell level. In this study, we provide definitive evidence of IgG secretion rates in early-minted ASC and mature BM ASC as well as early-minted ASC that have undergone *in vitro* maturation.

We demonstrate that ASC maturation is accompanied by both elevated Ig transcript levels (8) and increased Ig secretion capacity, highlighting the enhanced antibody-producing potential of mature BM plasma cells. Our results contradict previous thoughts that blood ASC had required rapid Ig production with high Ig secretion rates in response to acute infections, and that mature BM plasma cells have lower and slower secretion rates to sustain long-term immunity. Our findings align with the model that in the BM niche, ASC maturation undergo transcriptional and/or post-transcriptional adaptations such as elevated Ig transcript levels in LLPC (8) to optimize their role as antibody-secreting factories. Moreover, our findings show that the ASC maturation process provides an explanation for the wide range of secretion rates by human ASC (12, 14, 17, 18, 28–30).

A key contribution of this study is the use of a novel *in vitro* BM mimetic survival system (10), which allowed us to track the maturation of ASC (8, 11) and quantify their IgG secretion dynamics over time. The finding that early-minted blood ASC cultured in this system for 7 days secreted more IgG per cell than their day 0 counterparts suggests that the factors in the BM microenvironment play a critical role in driving functional maturation. This increase in Ig secretory capacity may reflect upregulation of Ig transcripts, enhanced ER function, or improved secretory pathway efficiency as ASC mature (31). These results underscore the importance of the BM niche in supporting not only plasma cell survival but also their functional specialization. This *in vitro* BM mimetic survival system offers a useful tool for studying human plasma cell biology, including ASC maturation *in vitro*.

The observed differences in IgG secretion rates between early-minted blood ASC and mature BM plasma cells have implications for understanding humoral immunity. Higher secretion rates in BM plasma cells likely contribute to the sustained antibody production required for long-term immune protection in response to vaccination or infection (7, 9). This efficiency of BM plasma cells to secrete nearly twice as much antibody per cell suggests prioritizing higher efficiency and fewer LLPC needed to achieve serologic protection. Conversely, the lower secretion rates of early-minted blood ASC may reflect their transient role in acute immune responses. These findings raise questions about the molecular mechanisms underlying the transition from early ASC to mature plasma cells, especially LLPC, including potential epigenetic or signaling pathways that regulate Ig secretion capacity.

While our study provides robust evidence for maturation-dependent increases in IgG secretion, certain limitations warrant consideration. First, the *in vitro* BM mimetic system, although effective in supporting ASC maturation, may not fully recapitulate the complexity of the *in vivo* BM niche, including interactions with cytokines or other immune cells. Second, our findings about lower IgG secretion rates in one particular myeloma cell line are specific to the ARH-77 cell line since no other myeloma cell lines or malignant human ASC were investigated. It is likely that secretion rates of other myeloma cell lines or primary myeloma cells are heterogeneous and vary significantly; and thus, one cannot conclude that myeloma cell lines or primary myeloma cells inherently secrete less antibody per cell compared to healthy ASC. Third, due to the limited cell numbers for evaluating phenotypic changes by flow cytometry, the frequencies of each BM ASC subset in cultures over time remain to be determined. Fourth, our focus on IgG secretion leaves open questions about whether similar trends apply to other Ig isotypes, such as IgA or IgM, which may be relevant in specific immune contexts. Finally, to address the detection limit of single-day bulk cultures and estimate the average secretion rates during ASC maturation, we calculated the total Ig secreted per cell per day over multiple days in bulk cultures. Although our 1-day single cell on-chip cultures confirm mature ASC have higher IgG secretion rates, this calculation does not account for variations in Ig production among different ASC over the course of multiple days. Future studies are needed to investigate the translational potential of modulating ASC maturation to enhance antibody responses in clinical settings, such as vaccine or immunotherapy.

In conclusion, our study highlights the functional specialization of human ASC as they mature into BM LLPC, with significant increases in per-cell IgG secretion rates. Our results are consistent with increased Ig transcripts of mature BM plasma cells (8) which results in higher Ig secretion rates. These findings advance our understanding of plasma cell biology and emphasize the critical role of the BM niche in optimizing antibody production. As LLPC are the basis of a successful vaccine (7), our findings emphasize the role of ongoing ASC maturation in the BM and highlight the importance of filling the BM LLPC compartment (9) for more stable and durable immunity. Further exploration of the molecular and environmental factors driving these changes will be essential for harnessing the full potential of ASC in therapeutic applications.

## Data Availability

The raw data supporting the conclusions of this article will be made available by the authors, without undue reservation.

## Supplementary material

Supplementary Figure 1 General FACS gating strategy used for sorting blood ASC and BM ASC. **(a)** PBMC or **(b)** BMMC were first gated for lymphocytes, singlets, and viable cells (based on FSC/SSC and Live/Death properties). CD3 and CD14 were then used as dump markers to capture CD19+ and CD19^-^ B cell populations. **(a)** Subsequent sub-gating using CD38 vs CD27 on the IgD^-^ fraction (of CD19^+^ population) allows for sorting for blood ASC (IgD^-^CD27^hi^CD38^hi^). **(b)** Subsequent sub-gating from CD19^+^ population on the IgD^-^ fraction (vs CD27) and using CD138 versus CD38 allowed for breaking down BM ASC populations into three subsets of interest: PopA (CD19^+^CD38^hi^CD138^-^), PopB (CD19^+^CD38^hi^CD138^+^), and PopD (LLPC; CD19^-^CD38^hi^CD138^+^).

Supplementary Figure 2 Summary of the techniques and experimental designs for detection of total IgG, Tet-IgG, and Flu-IgG ASC, as well as IgG secreted in the culture supernatants by ELISpots and the Lightning platform, as well as by ELISA and MBBA, respectively. Tet, tetanus. Flu, influenza.

Supplementary Figure 3 Cumulative total IgG and Tet-IgG by early-minted blood ASC maintained in bulk cultures for up to 7 days. Each symbol represents one experiment (per each timepoint). Numbers (n) indicate the quantity of independent biological experiments. For statistical differences among timepoints, see **Supplementary Table 3**.

Supplementary Figure 4 Total IgG and Flu-IgG secretion rates by blood ASC maintained in single-cell cultures for 7–8 days. Each symbol represents one cell. The number (n) indicates the quantity of individual cells. R and *p* values calculated from simple linear regression analysis in GraphPad Prism (GraphPad Software) of data generated from 114 cells (out of individual 192 cells) positive for both total IgG and Flu-IgG. Flu, influenza.

## References

[1] Tarlinton DM Ding Z Tellier J Nutt SL. Making sense of plasma cell heterogeneity. Curr Opin Immunol. (2023) 81:102297. doi: 10.1016/j.coi.2023.102297, PMID: 36889029

[2] Fooksman DR Jing Z Park R. New insights into the ontogeny, diversity, maturation and survival of long-lived plasma cells. Nat Rev Immunol. (2024) 24:461–70. doi: 10.1038/s41577-024-00991-0, PMID: 38332373

[3] Nguyen DC Saney C Hentenaar IT Cabrera-Mora M Capric V Woodruff MC. Majority of human circulating IgG plasmablasts stop blasting in a cell-free pro-survival culture. Sci Rep. (2024) 14:3616. doi: 10.1038/s41598-024-53977-2, PMID: 38350990 PMC10864258

[4] Garimalla S Nguyen DC Halliley JL Tipton C Rosenberg AF Fucile CF. Differential transcriptome and development of human peripheral plasma cell subsets. JCI Insight. (2019) 4(9):e126732. doi: 10.1172/jci.insight.126732, PMID: 31045577 PMC6538338

[5] Halliley JL Tipton CM Liesveld J Rosenberg AF Darce J Gregoretti IV. Long-lived plasma cells are contained within the CD19(-)CD38(hi)CD138(+) subset in human bone marrow. Immunity. (2015) 43:132–45. doi: 10.1016/j.immuni.2015.06.016, PMID: 26187412 PMC4680845

[6] Slifka MK Antia R Whitmire JK Ahmed R. Humoral immunity due to long-lived plasma cells. Immunity. (1998) 8:363–72. doi: 10.1016/S1074-7613(00)80541-5, PMID: 9529153

[7] Amanna IJ Carlson NE Slifka MK. Duration of humoral immunity to common viral and vaccine antigens. N Engl J Med. (2007) 357:1903–15. doi: 10.1056/NEJMoa066092, PMID: 17989383

[8] Duan M Nguyen DC Joyner CJ Saney CL Tipton CM Andrews J. Understanding heterogeneity of human bone marrow plasma cell maturation and survival pathways by single-cell analyses. Cell Rep. (2023) 42:112682. doi: 10.1016/j.celrep.2023.112682, PMID: 37355988 PMC10391632

[9] Nguyen DC Hentenaar IT Morrison-Porter A Solano D Haddad NS Castrillon C. SARS-CoV-2-specific plasma cells are not durably established in the bone marrow long-lived compartment after mRNA vaccination. Nat Med. (2024) 31(1):235–244. doi: 10.1101/2024.03.02.24303242, PMID: 39333316 PMC11750719

[10] Nguyen DC Garimalla S Xiao H Kyu S Albizua I Galipeau J. Factors of the bone marrow microniche that support human plasma cell survival and immunoglobulin secretion. Nat Commun. (2018) 9:3698. doi: 10.1038/s41467-018-05853-7, PMID: 30209264 PMC6135805

[11] Joyner CJ Ley AM Nguyen DC Ali M Corrado A Tipton C. Generation of human long-lived plasma cells by developmentally regulated epigenetic imprinting. Life Sci Alliance. (2022) 5(3):e202101285. doi: 10.26508/lsa.202101285, PMID: 34952892 PMC8739272

[12] Hibi T Dosch HM. Limiting dilution analysis of the B cell compartment in human bone marrow. Eur J Immunol. (1986) 16:139–45. doi: 10.1002/eji.1830160206, PMID: 2869953

[13] Kometani K Nakagawa R Shinnakasu R Kaji T Rybouchkin A Moriyama S. Repression of the transcription factor Bach2 contributes to predisposition of IgG1 memory B cells toward plasma cell differentiation. Immunity. (2013) 39:136–47. doi: 10.1016/j.immuni.2013.06.011, PMID: 23850379

[14] Corti D Lanzavecchia A. Efficient methods to isolate human monoclonal antibodies from memory B cells and plasma cells. Microbiol Spectr. (2014) 2(5). doi: 10.1128/microbiolspec.AID-0018-2014, PMID: 26104354

[15] Eyer K Doineau RCL Castrillon CE Briseno-Roa L Menrath V Mottet G. Single-cell deep phenotyping of IgG-secreting cells for high-resolution immune monitoring. Nat Biotechnol. (2017) 35:977–82. doi: 10.1038/nbt.3964, PMID: 28892076

[16] Lanzavecchia A. Dissecting human antibody responses: useful, basic and surprising findings. EMBO Mol Med. (2018) 10(3):e8879. doi: 10.15252/emmm.201808879, PMID: 29363490 PMC5840544

[17] Bromage E Stephens R Hassoun L. The third dimension of ELISPOTs: quantifying antibody secretion from individual plasma cells. J Immunol Methods. (2009) 346:75–9. doi: 10.1016/j.jim.2009.05.005, PMID: 19465022

[18] Pinna D Corti D Jarrossay D Sallusto F Lanzavecchia A. Clonal dissection of the human memory B-cell repertoire following infection and vaccination. Eur J Immunol. (2009) 39:1260–70. doi: 10.1002/eji.200839129, PMID: 19404981 PMC3864550

[19] Werner-Favre C Matthes T Barnet M Zubler RH. High IgE secretion capacity of human plasma cells. Eur J Immunol. (1993) 23:2038–40. doi: 10.1002/eji.1830230849, PMID: 8344372

[20] Salmon SE Smith BA. Immunoglobulin synthesis and total body tumor cell number in IgG multiple myeloma. J Clin Invest. (1970) 49:1114–21. doi: 10.1172/JCI106327, PMID: 4987170 PMC322579

[21] Nguyen DC Duan M Ali M Ley A Sanz I Lee FE. Plasma cell survival: The intrinsic drivers, migratory signals, and extrinsic regulators. Immunol Rev. (2021) 303:138–53. doi: 10.1111/imr.13013, PMID: 34337772 PMC8387437

[22] Shihan MH Novo SG Le Marchand SJ Wang Y Duncan MK. A simple method for quantitating confocal fluorescent images. Biochem Biophys Rep. (2021) 25:100916. doi: 10.1016/j.bbrep.2021.100916, PMID: 33553685 PMC7856428

[23] Liu X Yao J Zhao Y Wang J Qi H. Heterogeneous plasma cells and long-lived subsets in response to immunization, autoantigen and microbiota. Nat Immunol. (2022) 23:1564–76. doi: 10.1038/s41590-022-01345-5, PMID: 36316480

[24] Koike T Fujii K Kometani K Butler NS Funakoshi K Yari S. Progressive differentiation toward the long-lived plasma cell compartment in the bone marrow. J Exp Med. (2023) 220(2):e20221717. doi: 10.1084/jem.20221717, PMID: 36515679 PMC9754767

[25] Robinson MJ Ding Z Dowling MR Hill DL Webster RH McKenzie C. Intrinsically determined turnover underlies broad heterogeneity in plasma-cell lifespan. Immunity. (2023) 56:1596–1612 e4. doi: 10.1016/j.immuni.2023.04.015, PMID: 37164016

[26] Underhill GH George D Bremer EG Kansas GS. Gene expression profiling reveals a highly specialized genetic program of plasma cells. Blood. (2003) 101:4013–21. doi: 10.1182/blood-2002-08-2673, PMID: 12543863

[27] Tarte K Zhan F De Vos J Klein B Shaughnessy J Jr. Gene expression profiling of plasma cells and plasmablasts: toward a better understanding of the late stages of B-cell differentiation. Blood. (2003) 102:592–600. doi: 10.1182/blood-2002-10-3161, PMID: 12663452

[28] Henn AD Rebhahn J Brown MA Murphy AJ Coca MN Hyrien O. Modulation of single-cell IgG secretion frequency and rates in human memory B cells by CpG DNA, CD40L, IL-21, and cell division. J Immunol. (2009) 183:3177–87. doi: 10.4049/jimmunol.0804233, PMID: 19675172 PMC2765874

[29] Cheng RY Hung KL Zhang T Stoffers CM Ott AR Suchland ER. Ex vivo engineered human plasma cells exhibit robust protein secretion and long-term engraftment *in vivo* . Nat Commun. (2022) 13:6110. doi: 10.1038/s41467-022-33787-8, PMID: 36245034 PMC9573882

[30] Corti D Voss J Gamblin SJ Codoni G Macagno A Jarrossay D. A neutralizing antibody selected from plasma cells that binds to group 1 and group 2 influenza A hemagglutinins. Science. (2011) 333:850–6. doi: 10.1126/science.1205669, PMID: 21798894

[31] Shaffer AL Shapiro-Shelef M Iwakoshi NN Lee AH Qian SB Zhao H. XBP1, downstream of Blimp-1, expands the secretory apparatus and other organelles, and increases protein synthesis in plasma cell differentiation. Immunity. (2004) 21:81–93. doi: 10.1016/j.immuni.2004.06.010, PMID: 15345222

