# Supplementary figures and images for: Maturation of human early-minted blood antibody-secreting cells is coupled with increased IgG secretion rates
Source: Front Immunol. 2025 Sep 2;16:1644102. doi: 10.3389/fimmu.2025.1644102 (PMC12439162; doi:10.3389/fimmu.2025.1644102)

Supplementary Figure 1

**a**

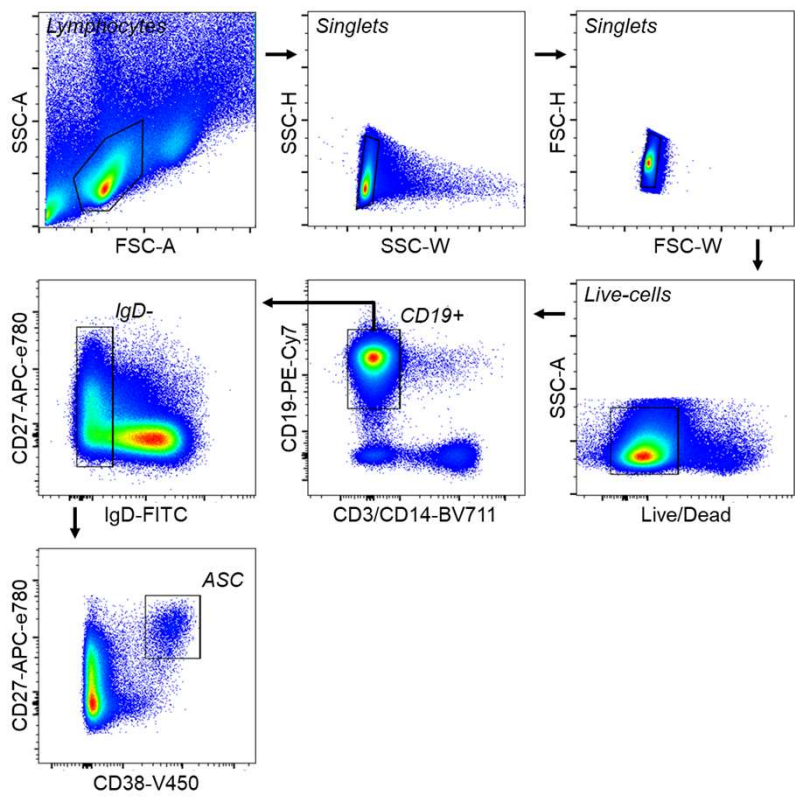

**b**

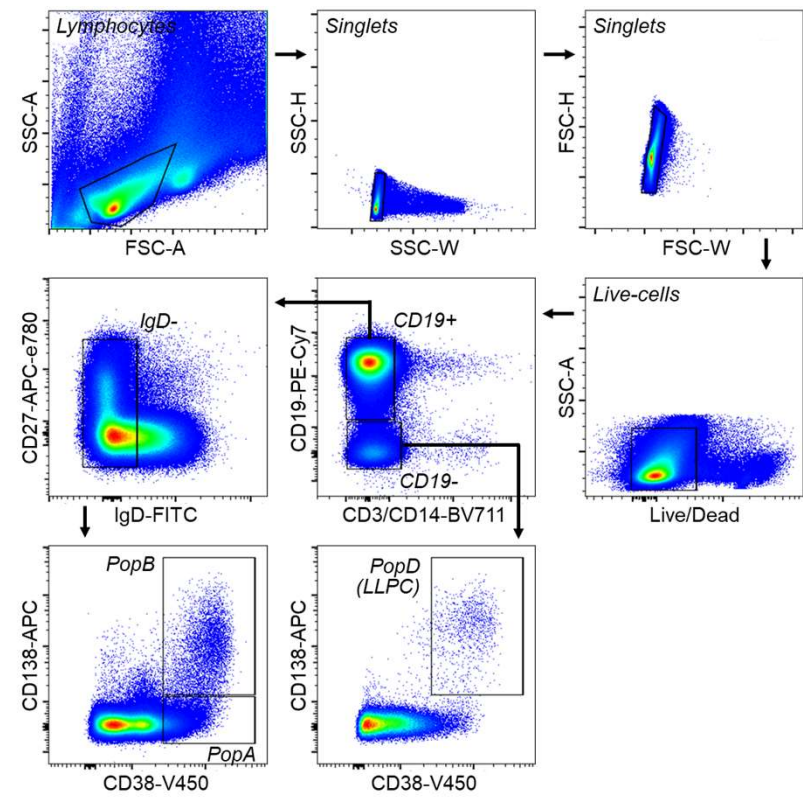

Supplementary Figure 2

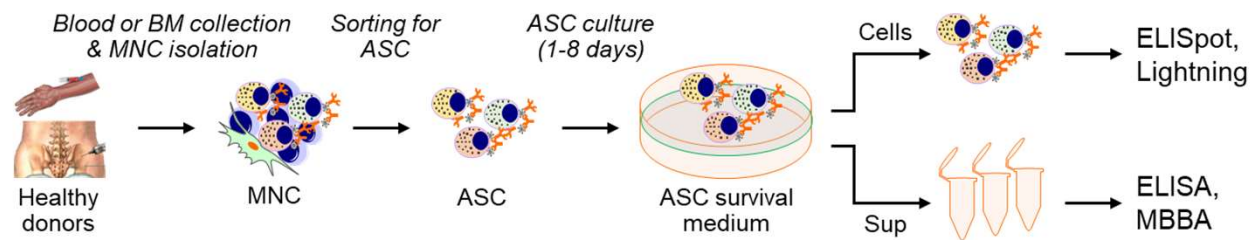

Supplementary Figure 3

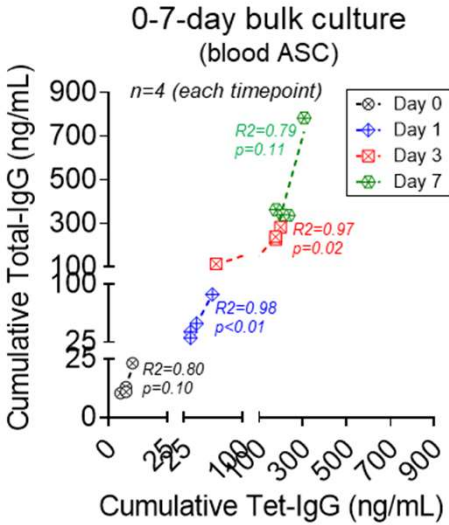

Supplementary Figure 4

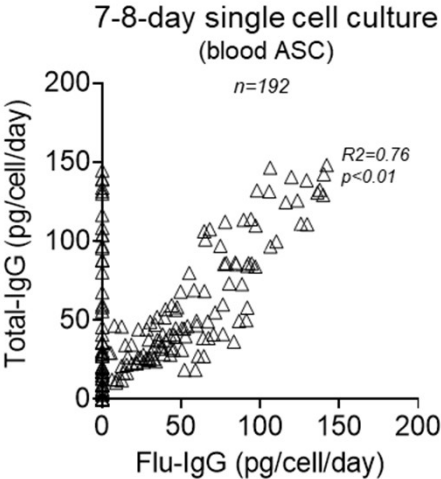

Supplement: Supplementary Figure 1 — General FACS gating strategy used for sorting blood ASC and BM ASC. (a) PBMC or (b) BMMC were first gated for lymphocytes, singlets, and viable cells (based on FSC/SSC and Live/Death properties). CD3 and CD14 were then used as dump markers to capture CD19+ and CD19- B cell populations. (a) Subsequent sub-gating using CD38 vs CD27 on the IgD- fraction (of CD19+ population) allows for sorting for blood ASC (IgD-CD27hiCD38hi). (b) Subsequent sub-gating from CD19+ population on the IgD- fraction (vs CD27) and using CD138 versus CD38 allowed for breaking down BM ASC populations into three subsets of interest: PopA (CD19+CD38hiCD138-), PopB (CD19+CD38hiCD138+), and PopD (LLPC; CD19-CD38hiCD138+). [file DataSheet1.pdf]
